# Detection of Urinary Antibodies and Its Application in Epidemiological Studies for Parasitic Diseases

**DOI:** 10.3390/vaccines9070778

**Published:** 2021-07-12

**Authors:** Fumiaki Nagaoka, Tatsuya Yamazaki, Sachiko Akashi-Takamura, Makoto Itoh

**Affiliations:** Department of Microbiology and Immunology, School of Medicine, Aichi Medical University, Aichi 480-1195, Japan; fnagaoka@aichi-med-u.ac.jp (F.N.); yamazaki13@aichi-med-u.ac.jp (T.Y.); sachiko@aichi-med-u.ac.jp (S.A.-T.)

**Keywords:** noninvasive test, urine diagnosis, urine ELISA, parasite infection, neglected tropical diseases (NTDs), mass drug administration (MDA)

## Abstract

For epidemiological studies of infectious diseases, pathogen-specific antibody levels in an area give us essential and appropriate information. The antibodies against pathogens are usually detected in blood, the drawing of which inconveniences people. Collection of blood increases the risk of accidental infections through blood, and it is difficult to obtain the participation of the target populations, especially the younger generation. On the other hand, urine samples, which contain a high enough level of antibodies for ELISA, can be harmlessly and easily collected and therefore have been used for epidemiological studies for diseases. The antibody examination of urine has been used for the epidemiology of parasitic diseases with a high sensitivity and specificity of serum samples. In this paper, we reviewed antibody assays with urine for seven parasitic diseases that urine diagnostic methods have reported in the past, and these are important infections included in NTDs, caused, for example, by *Leishmania donovani*, *Wuchereria bancrofti*, *Schistosoma japonicum*, *Paragonimus westermani*, *Echinococcus granulosus*, *Echinococcus multilocularis*, *Strongyloides stercoralis*, and *Opisthorchis viverrini*. The easy and safe urine surveillance system might be an admirable tool for future epidemiological studies for infectious diseases.

## 1. Introduction

Levels of pathogen-specific antibodies are useful information in epidemiological studies to determine the extent of the distribution of a pathogen and the intensity of its infection, to assess infection control programs, and to monitor for relapse. However, when the symptoms caused by the infection are mild and the rate of serious illness is low, or when the infection is no longer a threat to residents due to the progress of infection control programs, residents are not interested in the infection, and it is difficult to obtain their cooperation in epidemiological studies using painful blood collection.

Noninvasively collectable urine samples contain many serum components [1], and antibodies are also included in them. Although the concentration of antibodies in urine is only about 1/4000 to 10,000 of that in serum [2,3], it is possible to measure antibodies quantitatively using an ELISA method (urine ELISA) [4]. For a general clinical urine test, it is recommended to use the first collected urine early in the morning; however, urine antibody levels have been found to be unaffected by the time of urine collection [4]. As the levels of antibodies in urine correlate with those of serum samples [5], serum samples for epidemiological studies with pathogen-specific antibodies can be replaced by urine samples which are safely, easily, and noninvasively collected.

Urine ELISA methods have been reported to detect specific antibodies against viruses and bacteria such as *Helicobacter pylori* [2,3,5], rubella virus (RV) [6], dengue virus [7], hepatitis C virus (HCV) [8], and human immunodeficiency virus-1 (HIV-1) [9] and also against parasites such as *Leishmania donovani* [10], *Wuchereria bancrofti* [4], *Schistosoma japonicum* [11], *Paragonimus westermani* [12], *Echinococcus granulosus* [13], *Echinococcus multilocularis* [14], *Strongyloides stercoralis* [15], and *Opisthorchis viverrini* [16]. These are almost all of the parasitic diseases for which antibody detection methods in urine have been reported. In this review, the significance and future prospects of practical urinary antibody testing, mainly on the parasite-specific antibody detection method using urine ELISA, are described.

## 2. Characteristics of Urine Examination for Parasite-Specific Antibodies

### 2.1. Efficacy of Antibody Examination in Epidemiological Studies

In epidemiological studies of parasitic diseases, highly sensitive, highly specific, inexpensive, and simple methods are required. Although parasitological tests have been conventionally used to detect the parasite itself for parasite diseases, their sensitivity is often low. The tests to detect parasite-derived antigens and parasite-specific antibodies are used to complement these drawbacks. The antigen tests suggest that parasites are presently infecting (active infection); however, these are not so sensitive to detect a small amount of parasite exposure. On the other hand, although it takes nearly one week after infecting parasites provoke antibody levels to be detected, and it is impossible to distinguish present and past infections, antibody tests are generally sensitive and can detect parasite exposure that is not positive with antigen tests [17].

Investigating the distribution and incidence of parasitic diseases is very important for their control, in addition to establishing treatment methods and measures to prevent the spread of infection [18]. In situations where a parasitic disease is widely prevalent, parasitological tests might be sufficient to determine its prevalence. However, the sensitivity of these methods might be below the detection limit in the case of parasites reduced by their control programs; as a result, a small amount of residual parasite might be missed in the endemic area. In this regard, antibody tests can detect even weak parasite exposure, and they are particularly effective for epidemiological studies in these situations [19].

Each concentration of IgG, IgM, IgA, and secretory IgA in urine has been reported [20]. Pathogen-specific IgG (or its subclass), which is the highest concentration antibody class, has been detected with many urine ELISA methods. In some reports, IgA is also used to estimate infection [6,7]. Although the detection of specific IgM antibodies in urine has also been attempted, it does not seem to be sufficiently sensitive or specific due to their low content [7,9,21].

### 2.2. Advantages of Antibody Examination Using Urine Samples

**Figure 1:**
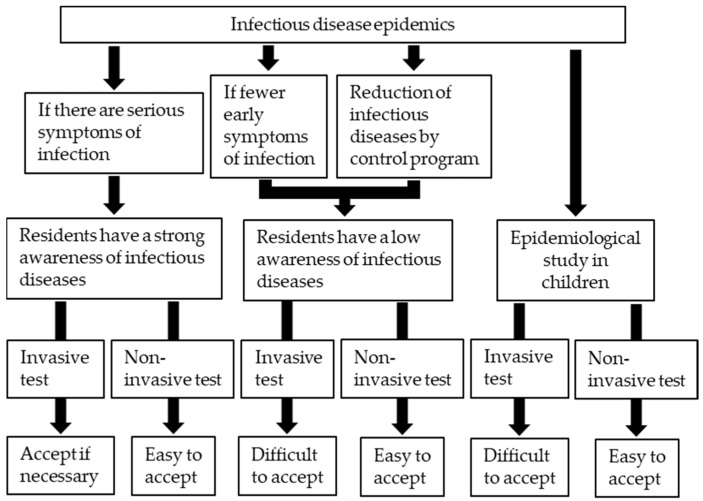
Characteristics of infectious diseases and tolerance of subjects to invasive and noninvasive tests in epidemiological studies.

There are three major advantages of using urine samples compared to serum in epidemiological studies. First, urine can be collected noninvasively. In general, antibody examinations use serum separated from blood samples. However, with blood sampling, it is difficult to obtain the cooperation of people due to the invasiveness, especially in situations in which the target population has less interest in the infectious disease, the epidemic is being contained by control programs, or the target population is children (Figure 1). For these reasons, noninvasive methods for epidemiological studies can easily obtain the cooperation of residents, and the urine-based antibody examination is the first candidate for this purpose. Second, urine can be collected at a low cost. Syringes, lancets, and deployment of medical personnel for blood collection are not required. Urine samples can be collected with the help of volunteers, such as villagers, schoolteachers, and even senior students; thus, study costs will be cut down. Third, the risk of accidental infection (e.g., hepatitis B virus, HCV, or HIV-1), which sometimes happens through blood, can also be greatly reduced.

## 3. Practical Examples of Application of Urine for Antibody Detection in Parasitic Diseases

The seven parasitic diseases described below are almost all of the reported cases of urine ELISA and are summarized in Table 1.

### 3.1. Visceral Leishmaniasis

Visceral leishmaniasis, caused, for example, by *L. donovani*, also known as kala-azar, is a zoonotic protozoan disease transmitted by the blood sucking of the sandfly. The protozoa is phagocytosed by macrophages in the human body and multiplies without being killed. *L. donovani*-infected macrophages circulate throughout the body, and many of them multiply rapidly in organs such as the liver, spleen, and bone marrow. On the other hand, some are taken into the sandfly by blood sucking and transmitted to new hosts. After infection and an incubation period of several months, hepatosplenomegaly, anemia, and fever appear. In addition, the infectious disease leads to death if untreated. Some of the patients suffer from skin lesions called post-kala-azar dermal leishmaniasis (PKDL) due to incomplete healing [22]. PKDL patients can also be a source of the parasite spreading, as the protozoa harbor in their skin lesions [23].

Definitive diagnostic methods for visceral leishmaniasis are to detect the parasites in biopsy samples of bone marrow, lymph nodes, and spleen. However, these methods are very invasive, and their usage is limited to hospitals with good facilities [24]. An ELISA and direct agglutination test (DAT), which detects *L. donovani*-specific antibodies in serum, have been used as complementary to them [25,26,27]. The immunochromatographic card test (ICT) with a recombinant antigen rK39 (Kalazar Detect^TM^ Rapid Test), which is derived from *Leishmania chagasi* kinesin-like protein, is sensitive and widely used for the diagnosis. It was reported that urine ELISA with acetone-treated *L. donovani* promastigote antigens for visceral leishmaniasis has a high sensitivity (95.0%) and specificity (95.3%) [10]. Higher sensitivity (97.94%) and specificity (100%) were obtained with *L. donovani* promastigote membrane antigens [28]. A recombinant antigen rKRP42, which is a part of *L. donovani* kinesin-related protein, was applied to the urine ELISA with a high sensitivity (94.0%) and specificity (99.6%) [29]. The comparison of three different recombinant antigens derived from *Leishmania* for the urine ELISA showed a high sensitivity and specificity with all antigens studied [30] (Table 1).

### 3.2. Lymphatic Filariasis

*W. bancrofti* and *Brugia malayi* are nematodes transmitted through mosquitoes. The adult worms mainly live in the lymphatic vessels and cause lymphangitis, lymphatic stagnation, scrotal edema and chyluria due to lymphatic vessel disruption, and elephantiasis [31]. These adult worms produce microfilariae (mf), which enter the bloodstream. However, generally, mf accumulate in the lungs during the day and mostly appear in peripheral blood at night, synchronized with the blood-sucking time of the vector mosquitoes [32].

Although the definitive diagnostic method is to detect mf in peripheral blood, the method is not very sensitive and often inconvenient because it can only be performed at night. An ICT kit (Binax Now^®^ Filariasis) detects a soluble antigen from *W. bancrofti* in blood. It has been used worldwide because of the high sensitivity and specificity and can be used during daytime. However, it is not quantitative [33].

The antibody titers will increase with even such a small exposure to the parasite; thereby, antibody examination is suitable to detect a history of infection among people in low endemic areas. A urine ELISA which detects IgG4 antibodies against *Brugia pahangi* crude antigens was developed with a sensitivity and specificity of 95.6% and 99.0%, respectively [4]. The method was found to be useful to evaluate a filariasis control program by mass drug administration (MDA), even in a low endemic area in Sri Lanka [34]. A recombinant antigen recWb-SXP1 from the *W. bancrofti* cDNA library was applied to the urine ELISA with a high sensitivity of 96.8% among mf positive and ICT positives, and of 84.8% among ICT positives, and a high specificity of 100% [35] (Table 1).

**Table 1:** Summary of antibody test for parasitosis by urine ELISA. mf: blood smear for microfilariae; Og4C3: ELISA kit to detect filarial circulating antigen; ICT: immunochromatographic card test for filarial antigen; SEN: sensitivity; SPE: specificity. * Percentage of urine antibody positive among serum antibody positive individuals.

Parasite Species	Antigen	Gold Standard	Detected Antibody	SEN (%)	SPE (%)	Sample Storage	Author, Year [Reference No]
*L. donovani*	acetone-treated *L. donovani* promastigote antigen	visceral leishmaniasis patients	IgG	95.0	95.3	4 °C	Islam et al., 2002 [10]
	*L. donovani* promastigote membrane antigen	visceral leishmaniasis patients	IgG	97.94	100	4 °C	Ejazi et al., 2016 [28]
	rKRP42 (recombinant antigen)	visceral leishmaniasis patients	IgG	94.0	99.6	4 °C	Islam et al., 2008 [29]
	rK28 (recombinant antigen)	visceral leishmaniasis patients	IgG	95.4	96.3	4 °C	Ghosh et al., 2016 [30]
	rK39 (recombinant antigen)			94.3	97.5		
	rKRP42 (recombinant antigen)			90.8	96.3		
*W. bancrofti*	adult female *B. pahangi* crude antigen	mf +/Og4C3 +	IgG4	95.6	99.0	4 °C	Itoh et al., 2001 [4]
	recWb-SXP1 (recombinant antigen)	mf +, ICT +	IgG4	96.8	100	4 °C	Samad et al., 2013 [35]
		mf +/-, ICT +		84.8			
*S. japonicum*	*S. japonicum* soluble egg antigen	serum antibody +	IgG	86.8 *	―	4 °C	Itoh et al., 2003 [11]
*P. westermani*	*P. westermani* adult worm crude antigen	paragonimiasis patients	IgG	100	―	4 °C	Qiu et al., 2016 [12]
			IgG4	90	―		
*E. granulosus* & *E. multilocularis*	hydatid antigen from cysts	cystic echinococcosis patients	IgG	84	76	−20 °C	Sunita et al., 2007 [13]
	hydatid antigen from cyst	cystic echinococcosis patients	IgG	80.48	93.75	−70 °C	Chirag et al., 2015 [21]
			IgM	48.78	100		
			IgG1	56.09	100		
			IgG2	53.65	100		
			IgG3	43.9	100		
			IgG4	65.85	100		
	*E. multilocularis* crude antigen from whole cysts	alveolar echinococcosis patients	IgG	83	99	4 °C	Itoh et al., 2013 [14]
			IgG4	91	98		
	recEm18 (recombinant antigen)		IgG	78	85		
			IgG4	78	100		
*S. stercoralis*	filariform *S. ratti* larva crude antigen	*S. stercoralis* + (agar plate culture)	IgG	92.7	40.7	4 °C	Eamudomkarn et al., 2018 [15]
*O. viverrini*	adult *O. viverrini* crude antigen	*O. viverrini* egg +	IgG	43.0	64.5	−70 °C	Tesana et al., 2007 [16]
			IgG4	45.9	67.2		

### 3.3. Schistosomiasis Japonica

*S. japonicum* is a zoonotic trematode of which the cercariae in a stagnant water area transdermally infect a host. In the human body, the cercariae develop to schistosomula, which enter the bloodstream and reach the hepatic portal vein, where they develop into adult worms. The eggs are produced in the blood vessel, and they cause emboli in the intestine and liver, and a part of them move into the feces through intestinal tissue [36]. The eggs are released into the environment with the feces, and miracidia in the eggs hatch in the water and invade shellfish, which are the intermediate host. In the shellfish body, miracidia develop into sporocysts to cercariae, which emigrate from the shellfish body and infect humans.

In the acute stage, some symptoms are observed such as diarrhea, abdominal pain, and viscous blood in the stool. In the chronic stage, liver cirrhosis and ascites accumulation are caused due to the embolization of worm eggs in the liver. It is a definitive diagnostic method to detect eggs discharged with tissue in stool, the sensitivity thereby being low. The detection of parasite-specific antibodies in serum has also been used as a method to compensate for stool examination [37,38]. A urine ELISA using a *S. japonicum*-soluble worm egg antigen was reported: 86.8% of those who were positive with serum antibody were positive with the urine ELISA [11] (Table 1).

### 3.4. Paragonimiasis

Paragonimiasis, which is a food-borne zoonotic disease, is transmitted by eating raw freshwater crab or animal meat containing *Paragonimus* spp. larvae [39]. The adult worms mainly live in the lungs, and the laid eggs are expelled with sputum. However, many of them are swallowed and excreted in feces. The discharged eggs hatch and grow into miracidia after about two weeks in a warm and humid environment. The miracidia invade shellfish, which are the first intermediate host. In the shellfish body, they develop into sporozoites, rediae, and cercariae. The cercariae emigrate from the shellfish and invade crabs, which are the second intermediate host, and form metacercaria.

The main symptoms are cough and bloody sputum. Although the laboratory test is to detect expelled eggs in sputum or feces, the sensitivity is low. In addition, some *Paragonimus* species do not grow to adult worms in human. In such cases, their eggs cannot be detected. Therefore, antibody examination is very useful for paragonimiasis. ELISA systems to detect specific antibodies in serum have already been developed [40,41]. A urine ELISA method has also been reported to detect the parasite-specific IgG against adult worms of *P. westermani* with a high sensitivity of 100%. Although the sensitivity became slightly low at 90%, measuring antigen-specific IgG4 antibodies can reduce cross-reactivity with other trematodes compared to IgG [12] (Table 1).

### 3.5. Echinococcosis

Echinococcosis is a zoonotic disease mainly caused by *E. granulosus* and *E. multilocularis*. People are infected by consuming water or foods contaminated with the eggs excreted from the final hosts, canids [42]. Humans play the role of intermediate host of the life cycle. Hexacanth embryos hatch from ingested eggs in the small intestine and invade the intestinal wall. The hexacanth embryos are transported to the whole body by hematogenous and lymphatic routes. Their hydatid cysts are mainly formed in the liver. In the environment, the eggs are ingested by intermediate hosts, such as goats or mice (depending on the worm species). The predation of these intermediate hosts results in the transmission of infection to the terminal host.

The infection is often asymptomatic, and it takes several years to become evident. As both *E. granulosus* and *E. multilocularis* do not grow to adults in humans, testing with eggs is not possible. The diagnosis is made by a combination of image diagnosis and serum antibody test using ELISA or Western blot. However, early diagnosis with these is not easy [43]. A comparative study of the sensitivity of parasite-specific antibodies in urine, blood, and saliva of patients showed a higher sensitivity with urine than two other specimens, and the specificity was equal among each sample [13]. A number of positive samples in each class and subclass of antibodies were compared in a urine ELISA; IgG was significantly higher than that of IgM, IgG1, IgG2, and IgG3, and there was no significant difference between IgG and IgG4. It was concluded that IgG, IgG1, and IgG4 in urine were the most important specific antibodies for the diagnosis of cystic echinococcosis [21]. Furthermore, it has also been reported that nonspecific responses in urine ELISA can be reduced by changing the antigen from cyst-derived crude antigen to recombinant antigen and the antibody to be measured from IgG to IgG4 [14] (Table 1).

### 3.6. Strongyloidiasis

Strongyloidiasis is one of the soil-borne nematode diseases caused by transdermal infection with filariform larvae of *S. stercoralis*. The adult worms of their nematodes live in the small intestine, where they lay the eggs. These larvae hatch in a few hours, and the larvae usually appear in the feces. These larvae develop into infective filariform larvae in the natural environment and infect humans.

The main symptoms are diarrhea and abdominal pain, and a small number of infections are often asymptomatic. In addition, an immunodeficiency caused, for example, by the administration of immunosuppressive agents, acquired immunodeficiency syndrome, and adult T-cell leukemia can lead to accelerated autoinfection in which the number of infective worms rapidly increases in the human body, resulting in serious illness [44]. The diagnosis is made by detecting larval nematodes in the feces and filariform larvae in stool cultures. The detection sensitivity of the culture method is relatively high, while the operation is complicated and there is a risk of infection by filarial larvae. Although some methods have been reported for the detection of *S. stercoralis*-specific antibodies in serum, these antibodies tend to be cross-reactive with other nematodes. The specificity varies greatly depending on the subject being infected by other nematodes [45]. A urine ELISA for strongyloidiasis was reported with crude antigens of filarial larvae of *Strongyloides ratti*; however, the specificity was not very high (40.7%) [15] (Table 1).

### 3.7. Opisthorchiasis

*O. viverrini* is a kind of liver fluke, which infects its host through the ingestion of raw or undercooked freshwater fish of *Cyprinidae* [46]. The adult worms of *O. viverrini* live in the bile ducts, and the eggs are produced in bile and discharged with stool. The eggs are ingested by a shellfish, which is the first intermediate host. Miracidia in the eggs hatch in the shellfish body and develop into sporocysts, rediae, and cercariae. The cercariae migrate from the shellfish and invade the fish, the second intermediate host, and they form metacercariae.

*O. viverrini* have been implicated in cholangiocarcinoma [47,48]. It was reported that anti-*O. viverrini*-specific antibody titers were significantly high in cholangiocarcinoma patients in *O. viverrini*-endemic areas [49]. Although stool examination to detect the eggs is generally used for the diagnosis, the detection sensitivity is low because of their low egg production. As alternative methods, detection of the parasite-specific antibodies has been attempted. However, the antibody response of the host is low in both serum and urine for examining the parasite infection because the larvae and adult worms do not invade host tissues [16] (Table 1). Further technological development is required to establish an immunodiagnostic method.

## 4. Application of Urine ELISA to the Sentinel Surveillance System

The seven parasitic diseases described above are the neglected tropical diseases (NTDs) for which control programs under the leadership of the WHO are ongoing [50]. Specific antibodies raised by past infections disturb the evaluation of recent infections. A younger age group, such as schoolchildren, is the suitable group as the sentinel for surveillance of new infections. It is difficult to obtain blood samples from this young group; however, urine samples are easily collected noninvasively. Elimination programs for lymphatic filariasis, MDAs, have been carried out by targeting all residents of endemic areas, regardless of whether they are infected or not [51]. Urine ELISA targeted toward school children in an endemic area in Sri Lanka showed a significant decrease in antibody levels and a positive rate as the MDA program progressed. These results indicate the usefulness of the sentinel surveillance system with this urine ELISA targeting school children [34].

## 5. Collection, Handling, and Storage of Urine Samples for Antibody Detection

**Figure 2:**
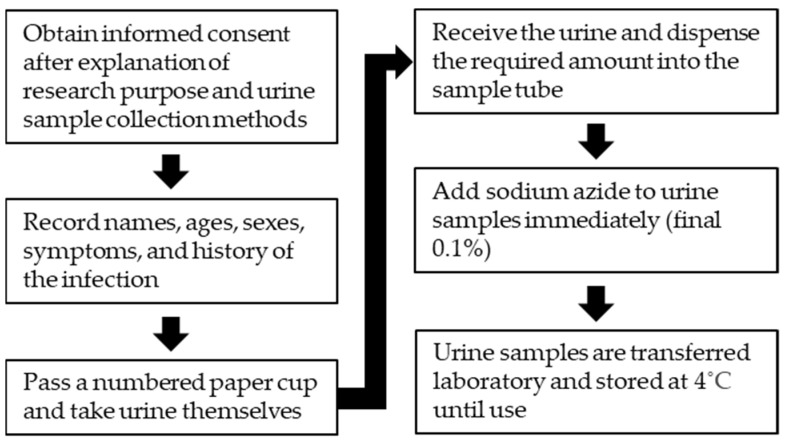
An example of a urine sample collection procedure.

An example of the urine sample collection flow is shown in Figure 2. The purpose of survey and the urine collection method are explained to people. After obtaining informed consent, they are asked their names, ages, sexes, disease histories, and presence of symptoms. Then, paper cups are passed out for them to collect their own urine samples. Even younger elementary school children can easily collect their urine, sometimes with the help of older students.

Although urine samples could be stored at frozen conditions in some reports [7,8,13], long-term freezing of urine samples caused false positives or false negatives [5] and decreased the sensitivity compared to unfrozen urine [52]. The effect of the storage term and temperature of urine samples for ELISA was investigated. In a report on the effect of storing urine at 37 °C, the results of the urine ELISA detecting anti-filarial IgG4 were not across the cutoff line in all 15 urine samples during the 4 weeks examined, although one positive sample showed a decrease in antibody titers [4]. Moreover, in *S. japonicum*-specific IgG, the levels of antibodies in urine were almost unaffected for 8 weeks of storage at 37 °C, and none of them crossed the cutoff value [11]. On the other hand, there are reports that IgG antibodies in urine were stable for at least 5 months when stored at 4 °C or 25 °C [53] and that IgG antibodies did not change for more than 18 months when stored at 4 °C [52].

Urine antibody detections were strongly interfered with by extremely low or high pH urine (data not shown). Therefore, urine samples for ELISA are commonly added to sodium azide to a final concentration of 0.1% (*w*/*v*) to avoid a change of urine pH as an effect of contamination and growth of bacteria [2,4,10,11,12,15,28,30,35,52]. However, as the addition of sodium azide does not completely inhibit the growth of bacteria [54], the development of new preservatives that keep the antibodies in urine stable for a long period is needed.

## 6. Discussion and Conclusions

In this review, we described ELISA systems for detecting parasite-specific antibodies in urine samples in seven parasitic diseases. The antibody diagnosis in urine might be a new standard tool for epidemiological studies in infectious diseases and is expected to be applied in the future.

Antibody levels of different isotypes and subclasses in urine might be used to estimate the time of infection and to increase the specificity. IgM is commonly used as an indicator of early infection in serum samples. However, its measurement is not suitable for urine samples due to sensitivity and specificity issues [7,9,21]. Rubella virus-specific IgA antibodies rise earlier than IgG in urine after RV vaccination, and the high IgA level is maintained for a while, whereas urinary RV-specific IgG rises slowly after vaccination. The urinary RV-specific IgA/IgG ratio peaks 4 weeks after vaccination, suggesting the possibility of early diagnosis of RV infection [6]. It is also interesting to find that the detection of parasite-specific IgG4 is superior in specificity to IgG with urine [12,14] as compared with serum [55,56,57] that is, detecting parasite-specific IgG4 might reduce a nonspecific reaction.

The usage of recombinant antigens for diagnosis ensures a stable supply of antigens for testing and can increase specificity [14,29,35]. The sensitivities and the specificities of urine ELISA for strongyloidiasis and opisthorchiasis, for which sufficient specificities and sensitivities have not been achieved, might be improved by careful selection of the recombinant antigens for the examination.

Urine ELISA is a highly sensitive and specific method for quantitative antibody detection in parasitic diseases. In addition, urine is more acceptable for its collection, especially from asymptomatic and healthy individuals and children, due to its less invasive nature, making it particularly useful for epidemiological studies of infectious diseases in these populations. The easy and safe urine surveillance system might be an admirable tool for future epidemiological studies for infectious diseases.
